# Influence of the Precursor, Molarity and Temperature on the Rheology and Structural Buildup of Alkali-Activated Materials

**DOI:** 10.3390/ma14133590

**Published:** 2021-06-27

**Authors:** Salman Siddique, Vivek Gupta, Sandeep Chaudhary, Solmoi Park, Jeong-Gook Jang

**Affiliations:** 1 Division of Architecture and Urban Design, Urban Science Institute, Incheon National University, 119 Academy-ro, Yeonsu-gu, Incheon 22012, Korea; salmansiddique@inu.ac.kr; 2 Department of Civil Engineering, Indian Institute of Technology Indore, Simrol, Indore 453552, India; phd1701104001@iiti.ac.in (V.G.); schaudhary@iiti.ac.in (S.C.); 3 Department of Civil Engineering, Pukyong National University, 45 Yongso-ro, Nam-gu, Busan 48513, Korea; solmoi.park@pknu.ac.kr

**Keywords:** fly ash, slag, alkali-activated material, rheology, loss factor, structural buildup

## Abstract

This study presents an investigation of the effects of the precursor, alkalinity and temperature on the rheology and structural buildup of alkali activated materials. Here, 100% fly ash, 100% slag and blended mixes of fly ash and slag were activated by 4 M, 6 M, 8 M or 10 M (only for sodium hydroxide) solutions at 25 °C, 35 °C, 45 °C and 55 °C. The rheological properties were investigated to obtain the flow curves, viscosity, storage modulus, and loss factor of these materials. The results showed that for the presence of slag, a higher molarity of the alkali activating solution and a high temperature all caused greater interparticle force, leading to an increase in the shear stress and viscosity of the alkali activated materials. It was also observed that slag had the greatest effect on the increase in the storage modulus of the blended mixes. Furthermore, the higher alkalinity and temperature levels were instrumental in initiating the dissolution of fly ash and improving its rate of structural buildup. Moreover, the interdependence of various factors showed that the type of precursor, as well as the concentration of alkali activating solution, were the primary influencing factors on the polymerization process, as well as the rheological measurements of alkali-activated materials.

## 1. Introduction

Alkali-activated materials have been identified as eco-friendly alternatives to ordinary Portland cement composites [1]. Furthermore, the superior performance of alkali-activated materials, in terms of the mechanical strength and durability, has been well established. Alkali-activated materials are often prepared by mixing a powder precursor and an alkaline solution, followed by a curing procedure [2]. Blast furnace slag and coal fly ash are the most commonly used precursors, followed by metakaolin and glass powder [3,4,5]. Binary blends of fly ash and slag mixes are also preferred given their ability to improve the fresh and mechanical properties of alkali-activated materials at room temperature [6,7,8,9]. The reaction kinetics of a binary blend of slag and fly ash have been widely reported as a gel structure consisting of calcium aluminate silicate hydrate (C-A-S-H) and sodium aluminosilicate hydrate (N-A-S-H) [10]. Furthermore, the physiochemical properties of alkali-activated materials are largely dependent on the ratio of the binary precursors, the molarity of the alkali solution, the type of alkali activator used, and the temperature. It should be noted that compared to ordinary Portland cement-based mixes, alkali-activated materials are prepared with an alkali-activated solution that has a higher viscosity than normal water. This, along with the faster reaction rate of an alkali-activated material, can provide a stark difference in rheological behavior as compared to that by Portland cement mix.

The reaction process of alkali-activated materials has been extensively studied using various characterization and microstructural techniques [1,11]. The three reaction stages of alkali-activated fly ash materials are: (1) dissolution of an aluminosilicate source into a solution; (2) gelation; and (3) the formation of a polymer reaction matrix due to the condensation of aluminates and silicates [12,13]. For alkali-activated slag: (1) the depolymerization of the alumino-silicate contained in raw materials into ions; (2) increase in precursor number and the enhancement of the reaction process; and (3) condensation and crystallization, which resulted in the formation of hydrated calcium alumino-silicate gels and hydrated calcium silicate gels [14]. It has been reported in limited studies that rheological measurements can be quite useful for understanding the reaction process of alkali-activated materials. Favier et al. reported that initial alumina-rich gel is responsible for an increase in the elastic modulus of alkali-activated metakaolin [12]. Palacios et al. observed that, when sodium hydroxide solution was used, slag-based alkali-activated mixes behaved as a Bingham fluid, showing shear thickening behavior [15]. Poulesquen et al. reported that when sodium hydroxide is used as an activator, the reaction kinetics are faster compared to the use of a potassium hydroxide-based activator [16]. However, alkali-activated materials based on potassium hydroxide present a more rigid microstructure network [16]. Rifaai et al. observed that excessive levels of an activator solution result in lower yield stress and storage modulus outcomes [17]. Ishwarya et al. concluded that when increasing the amount of slag in alkali-activated fly ash mixes, the yield stress also increases [18]. Dai et al. reported that a higher water-to-binder ratio was instrumental in increasing the strength as compared to a lower water-to-binder ratio for blended alkali-activated mixes [19]. Moreover, the slag portion of the blended mix made the main contribution to the gain in the storage modulus of alkali-activated material [19]. Palacios et al. found that temperature is quite influential in promoting the formation of reaction products in alkali-activated fly ash [20]. Temperatures above 65 °C led to an increase in the yield stress and the apparent viscosity values of alkali-activated mixes [20]. Vance et al. reported that the concentration of an alkali-activating solution had a strong influence on the viscosity of fly ash alkali-activated mixes [21]. It should be noted that alkali-activated materials are prepared from a wide spectrum of highly versatile precursors, such as slag, fly ash, and metakaolin. These precursors play a major role in the rheology of alkali-activated materials. In general, slag-based alkali-activated materials present higher initial fluidity levels than alkali-activated fly ash materials. However, alkali-activated slag usually shows a shorter setting time due to solidification and the formation of C-S-H, affecting the rheology [19].

Several previous studies have also reported that the rheological properties and structural buildup of an alkali-activated material are greatly influenced by the types of precursor and the activator used [19,20]. Despite the advances made by these studies, knowledge of the rheology of alkali-activated materials remains limited. Previous studies focused on single influencing factors on the rheology of alkali-activated materials. This approach is quite limited to provide a full scope of factors that can affect the rheology of alkali-activated materials. The influence of all of the aforementioned factors on the rheology, as well as the structural buildup, are also quite limited. This is also true with regard to the effects of various factors on the rheology of fly ash, slag, and blended alkali-activated mixes. A precise knowledge of the impact of various factors and conditions on the rheology of alkali-activated materials is therefore needed to understand the structural buildup. The present study aims to investigate the effects of several factors, specifically the type of precursor, the alkali-activating solution, and temperature on the rheological behaviors of alkali-activated materials. Specific attention was directed toward the effects of the above-mentioned factors on the rheology, reaction process, and structural buildup of alkali-activated materials over time. Moreover, the interdependence of various factors among each other, as well as influence of these factors on the reaction process of precursors from rheological measurements was evaluated.

## 2. Materials and Methods

### 2.1. Materials and Mix Details

Table 1 shows the chemical compositions (determined by X-ray fluorescence, PW 2404, Phillips, New Delhi, India) of blast furnace slag (obtained from a local industrial source in India, NICE, Surat) and fly ash (obtained from a coal-fired thermal power plant in India, NTPC, Nashik) used in the study. Using a 90-micron sieve, 95.0% of the fly ash particles passed, and this rate was 99.5% for the slag. The Blaine fineness of the fly ash was 320 m^2^/kg and it was 510 m^2^/kg for slag. Four different alkali-activating solutions were prepared by mixing 4M, 6M, 8M, and 10M sodium hydroxide and sodium silicate solution in a mass ratio of 1:1. The chemical composition of sodium silicate was SiO_2_ = 28 wt.%, Na_2_O = 9 wt.%, and H_2_O = 63\ wt.%. Furthermore, it should be noted that the 4M, 6M, 8M, and 10M indicate only the molarity of sodium hydroxide solution. A 24-h cooling period for the alkali-activating solution was considered before the preparation of the alkali-activated mixes. A constant liquid-to-precursor ratio of 0.45 for alkali-activated mixes was maintained throughout the study. Four different mixes were utilized: 100% fly ash (FA), 70% fly ash + 30% slag (70FA30S), 70% slag + 30% fly ash (70S30FA) and 100% slag (S). Paste samples were prepared in a Hobart mixer. The precursor powder was added to the activator solution in a mixing bowl, and mixed at low (140 ± 5 rpm) and high (285 ± 5 rpm) speeds, for 90 s each.

### 2.2. Rheological Measurements

Dynamic shear rheological measurements were carried out using an Anton Paar MCR 302 rheometer (Anton paar, New Delhi, India). The flow curves of the alkali-activated materials were obtained at a constant temperature of 25 °C. To measure the flow curves, a ball measuring system with ball size of 15 mm in diameter was employed. Rheological measurements were carried out as per the following protocol: (1) initial pre-shear at 100 s^−1^ for 30 s; (2) increase in the shear rate from 0 to 100 s^−1^ for 71 s; (3) decrease in the shear rate from 100 to 0 s^−1^ for 71 s; (4) a constant shear rate of 1 s^−1^ for 30 s; and (5) repeat of steps (2)–(3). The yield stress and plastic viscosity of the alkali-activated mixes were calculated using the Herschel–Buckley (HB) model, as shown in Equation (1).

(1) $$\tau =\tau_{0}+a\;\gamma^{b}$$

where *τ* is the shear stress in Pa, *τ_0_* is the yield stress in Pa, *γ* is the shear rate in 1/s, *a* is the consistency index, and *b* is the power law index.

For linear viscoelastic resonance (LVER) measurements, a constant frequency of 1 Hz was considered. The range of strain where G’ remained constant was considered as the LVER; i.e., elastic behavior is exhibited by the alkali-activated material. Here, γ_c_ is defined as the LVER limit and a 10% reduction of G’ is used to determine the γ_c_ value [22,23]. The LVER method is preferable as it simplifies the identification of the changes in G’ [24]. Here, G’ is defined as the storage modulus, which represents the elastic energy stored in the viscoelastic material, and G” is the loss modulus, which represents the energy consumed due to the viscous component of the viscoelasticity [25]. The LVER, storage and loss factor observations were done by employing a parallel plate arrangement. The parallel plate arrangement consisted of a pair of 25 mm abrasive plates. The storage and loss factor measurements were done for 4, 6, 8, and 10 molarity alkali-activating solutions at 25, 35, 45, and 55 °C. The temperature was controlled using a Peltier system attached to a rheometer. The parallel plate arrangement has been used in multiple studies to evaluate the rheological properties of cement composites [26,27], as well as those of alkali-activated and geopolymer composites [28,29].

## 3. Results

### 3.1. Flow Curves

**Figure 1:**
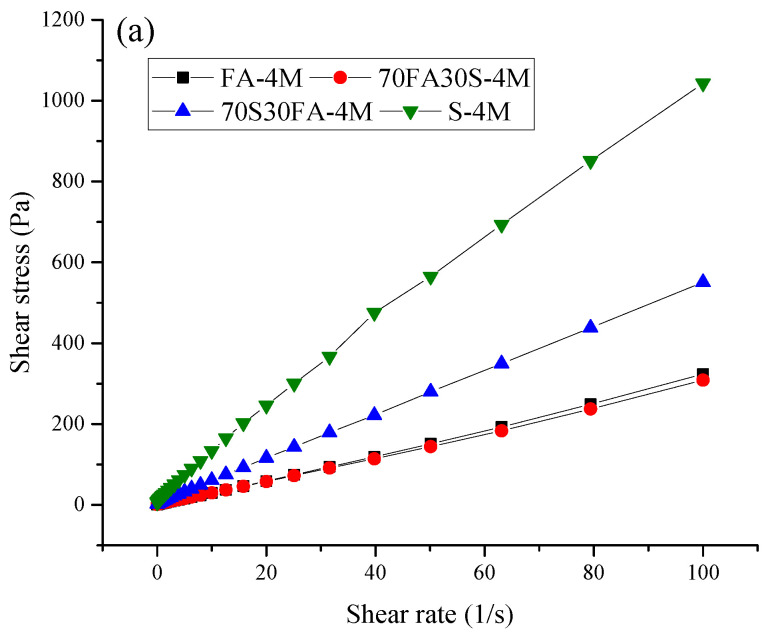

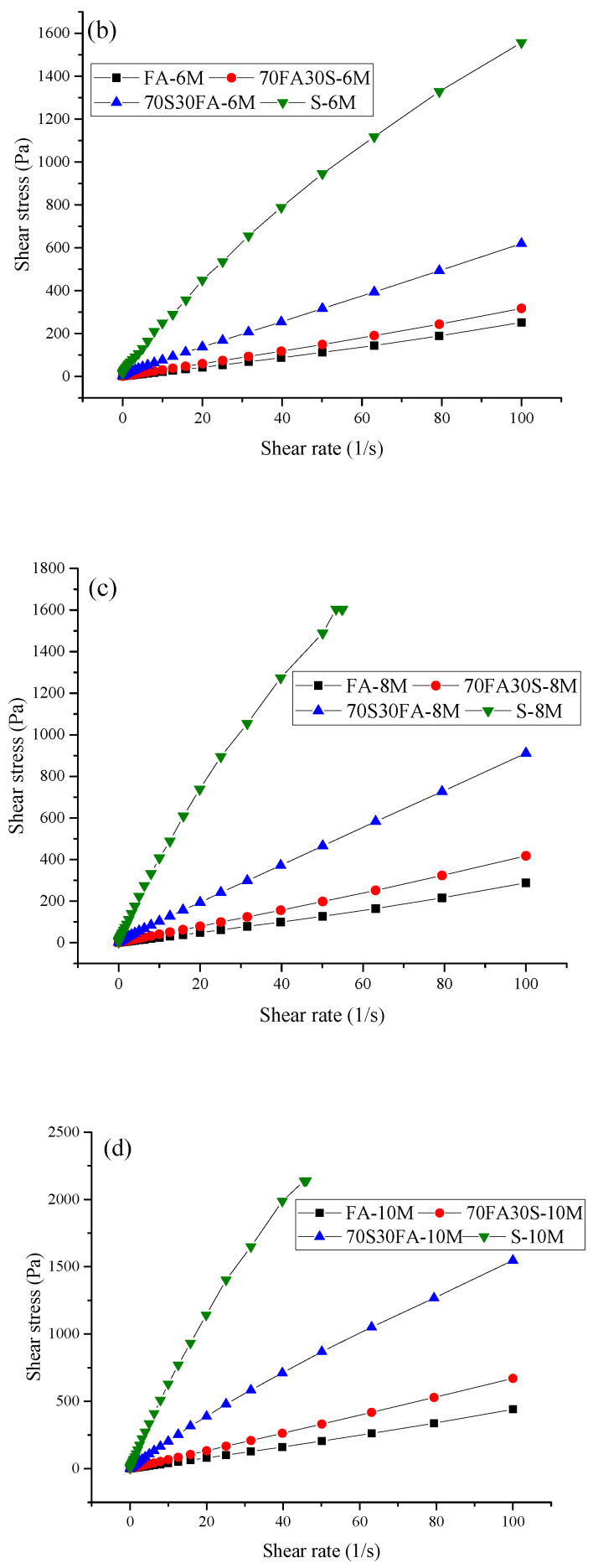
Flow curves for alkali-activated mixes: NaOH molarity of (**a**) 4 M, (**b**) 6 M, (**c**) 8 M, and (**d**) 10 M.

Figure 1 shows the flow curves of the alkali-activated materials. In addition, the dynamic yield stress values are presented in Table 2. It can be seen that the slag-based alkali activated mixes showed higher shear stress levels as compared to the blended mixes and alkali-activated fly ash. It should be noted that the temperature, humidity, and liquid-to-binder ratio were kept constant during the flow curve measurements. This indicates that the molarity of the alkali-activating solution and the physical/chemical properties of the precursor were responsible for the changes in the shear stress. The different reaction kinetics of the alkali-activated materials result in fairly different flow behaviors as compared to that of ordinary Portland cement [30]. It has been pointed out that the fluidity of alkali-activated paste is based on the fluid viscosity, interparticle forces, probable particle jamming and on the formation of a polycondensation product skeleton [21,31,32,33]. Given that the slag particles used here are finer than the fly ash in this study, the jamming of particles will influence the shear stress of the alkali-activated paste. Shear deformation of the alkali-activated material leads to mechanical interactions and particle restructuring [34]. Deformation into a mechanical state predominately results in the jamming of particle grains, which presents abrupt changes in the internal forces and in a release of the strain energy [35]. The finer particles of slag, therefore, recorded higher shear stress rates at a lower shear rate.

### 3.2. Viscosity Curves

In addition, it should be noted that, upon an increase in the content of slag in the alkali-activated fly ash paste, the reaction kinetics changed. The finer particle size along with the fast reaction rate of slag caused the rapid absorption of the alkali-activating solution and enhanced the formation of flocculants. As the amount of free liquid decreases, the shear stress increases. With regard to the alkali-activated fly ash, the fly ash particles are usually larger when compared to slag particles. This lowers the surface area, and less water is thus required to wrap particle surfaces. This results in a higher free liquid content and a lower solid volume fraction. Furthermore, the reactivity rate of fly ash has been found to be lower as compared to that of slag, which will decelerate flocculation and lowers the rate of polymerization, leading to a higher free liquid content. Another factor is the entrapment of air bubbles between the fly ash particles, due to the larger particle size of fly ash. Small air bubbles can provide a lubrication and ball bearing effect, which could improve the flowability of alkali-activated material [36].

Regarding the molarity of the alkali-activating solution, the interparticle force between NaOH and Na_2_SiO_3_ may also have an effect on the shear stress [20]. In the alkali-activating solution with lower molarity, the silicate ions of Na_2_SiO_3_ increase the negative surface charge of the precursor. The adsorption of silicate ions in to the precursor (fly ash or slag) results in increased interparticle repulsive forces, which cause an increase in the negative surface charge. The increased repulsion between the particles leads to deflocculation of the particles and, thus, a lower level of shear stress [37]. However, a higher molarity is indicative of an increased amount of NaOH in the alkali-activating solution, which results in a greater negative charge of the precursor particles, causing a significant repulsive force, which increases the shear stress of the alkali activated paste. Moreover, the higher molarity of the alkali-activating solution accelerates the reaction rate of the precursor [38,39]. Similar observations were drawn by Rifaai et al. [17] upon studying the influence of sodium hydroxide on the rheology of fly ash-based geopolymer. It was reported that the rheological polymerization process is greatly affected by the concentration of alkali activator [17].

**Figure 2:**
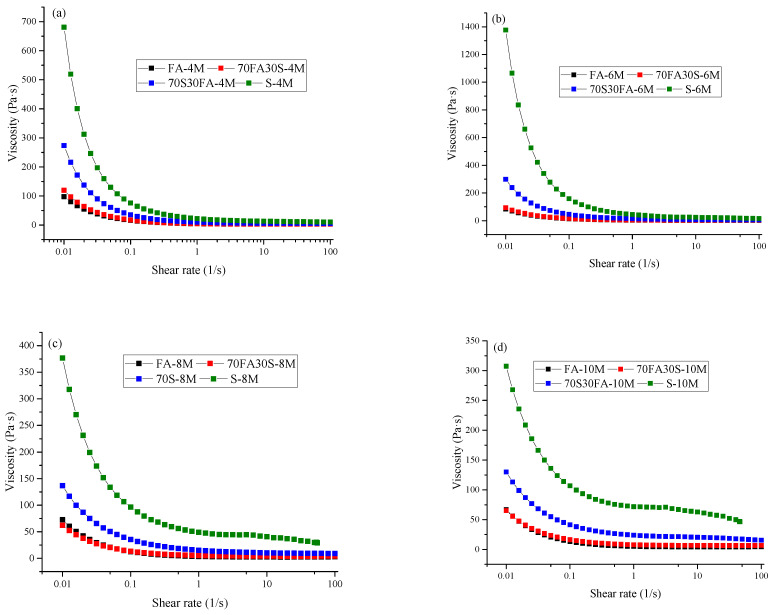
Viscosity curves for alkali activated mixes: NaOH molarity of (**a**) 4 M, (**b**) 6 M, (**c**) 8 M and (**d**) 10 M.

Figure 2 shows the viscosity of the alkali-activated material over time. In addition, a summary of the plastic viscosity values is presented in Table 2. The types of precursor and alkali-activating solution used were found to affect the viscosity of the alkali-activated paste. Furthermore, the reaction process of the precursor played an important role in the viscosity of the alkali-activated paste.

Viscosity is defined as resistance to a flow once the paste is in motion. It was observed that the slag-based alkali-activated paste here recorded a higher viscosity compared to that of the fly ash-based alkali-activated paste. Particle flocculation, governed by the shape and particle size distribution of the precursor, is one of the primary causes of viscous behavior. A finer particle size of slag, which promotes particle jamming, enhances the viscosity of the alkali-activated paste. Particle jamming is a physical process in which the viscosity of materials increases due to increased particle density. In addition, to some extent, the degree of dissolution of the particles, along with the reactivity of the different precursors, influence the viscosity. The slow reactivity of fly ash in developing alkali-activated materials and Portland cements has been widely reported in the literature [20,40]. In addition, increasing the amount of slag when replacing fly ash results in significant changes in the reaction products and the physical–chemical interactions. Slag enhances the breakdown and release of silicate and aluminate monomers. This results in a steady increase in viscosity, as shown in Figure 2.

It can also be observed in Figure 2 that a change in the molarity of the alkali-activating solution has an important effect on the viscosity of the alkali-activated paste. Figure 2 shows that an increase in the molarity of the activator solution leads to a higher viscosity over time. This demonstrates a directly proportional relationship between the OH^-^ concentration in the alkali-activating solution and the dissolution of the fly ash, which results in increased viscosity of the alkali-activated paste. It is also known that Na_2_SiO_3_ delays the viscosity onset time of alkali-activated paste. In the present study, when increasing the molarity of the alkali solution, the effect of Na_2_SiO_3_ is diluted, which accelerates and increases the viscosity of the alkali-activated paste. A proper analysis and investigation should be carried out to determine the appropriate balance between the alkali-activating solutions to provide insight into the rheological and physical properties of an alkali-activated paste. Similarly, a previous study showed that higher concentrations of activator lead to increased viscosity due to the formation of higher reaction products [20].

### 3.3. Linear Viscoelastic Range

**Figure 3:**
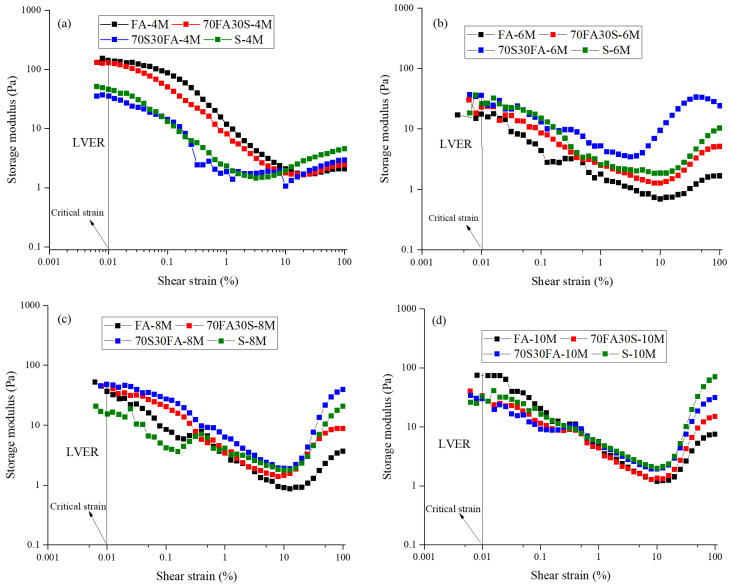
Evolution of storage modulus with the shear strain: NaOH molarity of (**a**) 4 M, (**b**) 6 M, (**c**) 8 M, and (**d**) 10 M.

The linear viscoelastic range (LVER) is defined as the strain range in which a viscoelastic material acts as an elastic material. Beyond this limit, the microstructure of the material is distorted and the material begins to act as a viscoelastic liquid [22,41,42]. In the present study, LVER serves as the strain range in which a constant storage modulus (G’) is observed (i.e., an elastic material). The limit of the LVER range is used to define γ_c_ and is considered as the strain corresponding to a 10% reduction in the storage modulus [22,23]. The LVER method is the preferred mode of investigation, as it can be used to identify a reduction in the storage modulus [24]. Figure 3 shows the LVER curves of the alkali-activated paste and Table 3 presents the LVER limit, LVER proposal, and storage modulus used in the present study. For the alkali-activated materials produced in the present study, the critical strain values are approximately 0.01%; however, for mixes with higher molarity levels, higher critical strain was observed—this was due to the higher repulsive forces between the precursor particles [43,44].

### 3.4. Storage Modulus

**Figure 4:**
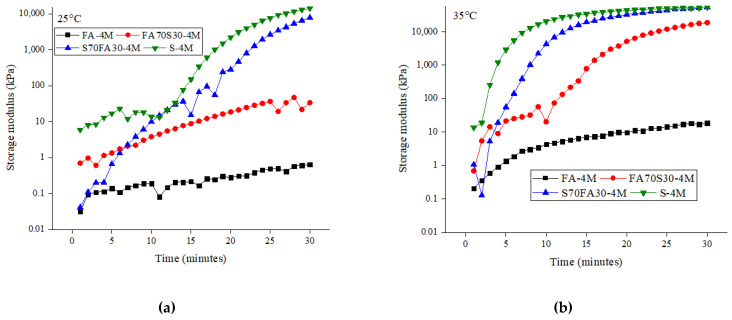

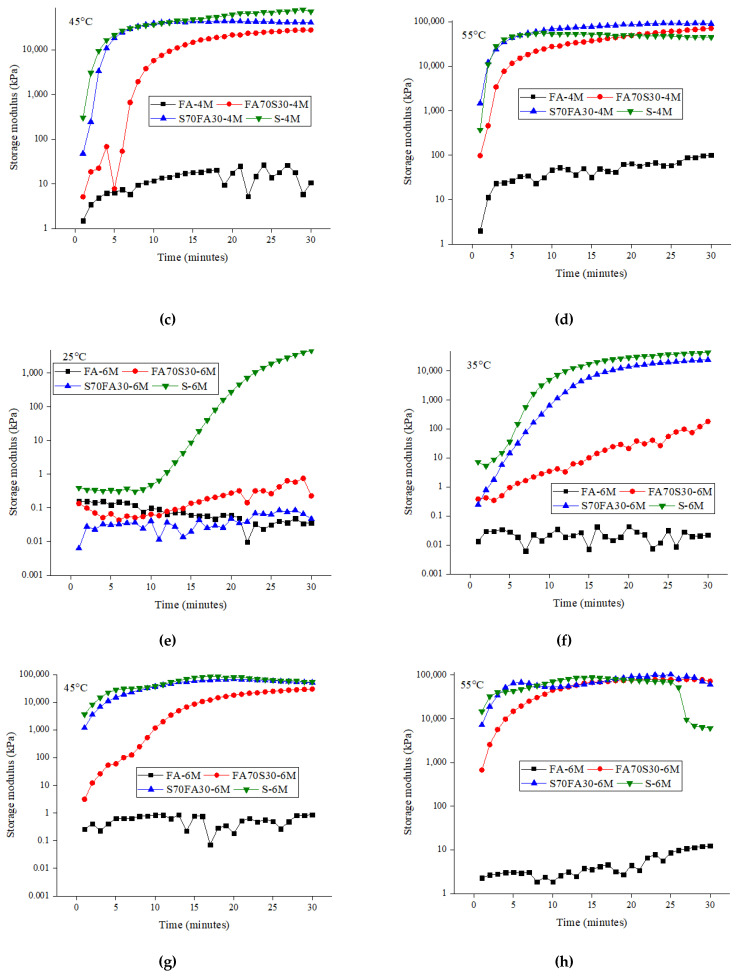

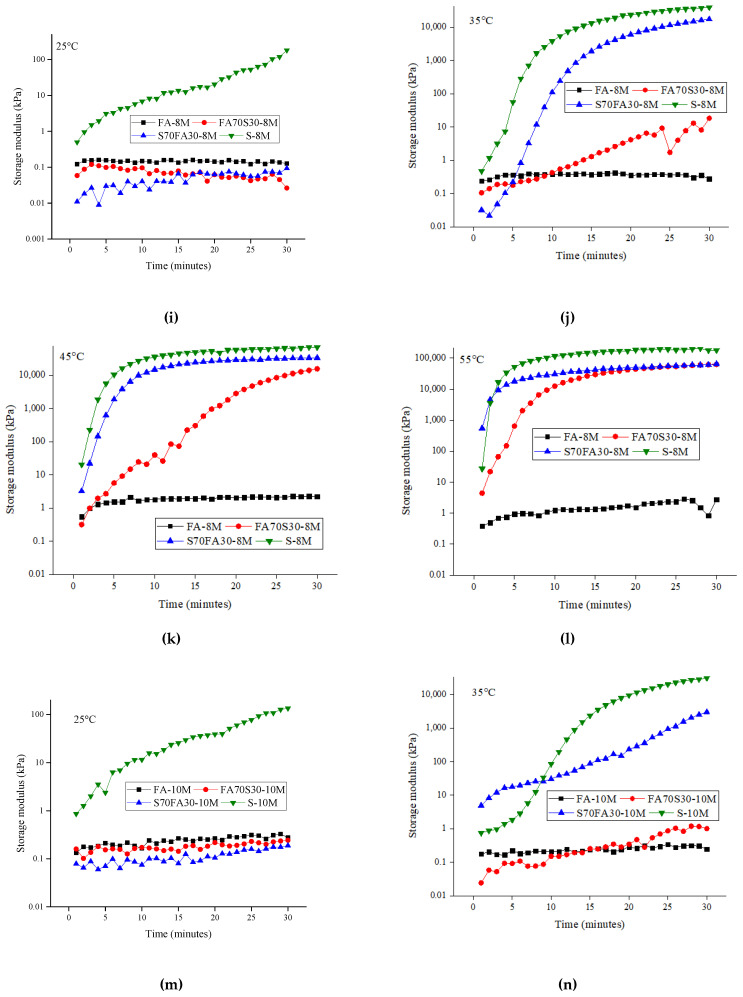

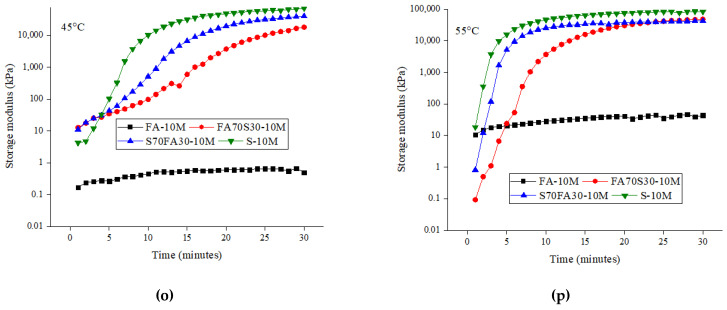
Evolution of storage modulus with time for alkali-activated mixes: (**a**) 4 M at 25 °C; (**b**) 4 M at 35 °C; (**c**) 4 M at 45 °C; (**d**) 4 M at 55 °C; (**e**) 6 M at 25 °C; (**f**) 6 M at 35 °C; (**g**) 6 M at 45 °C; (**h**) 6 M at 55 °C; (**i**) 8 M at 25 °C; (**j**) 8 M at 35 °C; (**k**) 8 M at 45 °C; (**l**) 8 M at 55 °C; (**m**) 10 M at 25 °C (**n**) 10 M at 35 °C (**o**) 10 M at 45 °C; (**p**) 10 M at 55 °C.

Figure 4 shows the storage modulus curves observed in the present study. The storage modulus values were observed at critical strain values of 0.01%. The influence of each parameter is discussed in detail below.

#### 3.4.1. Effect of Precursor

It can be observed in Figure 4 that the precursor plays an important role in determining the storage modulus of an alkali-activated paste. Alkali-activated slag shows higher growth rates and greater gains in the storage modulus as compared to alkali-activated fly ash and blended alkali-activated mixes. The van der Waals attraction forces are responsible for the formation of a flocculated microstructure, which influences the viscoelastic responses of binder pastes [45]. In addition, the rapid rate of the dissolution of ions also contributes to faster structural development [46]. For Portland-cement-based binders, it has been shown that the storage modulus during an early age shows a continuous increase, as early-age hydration products form small bridges between the cement particles [43]. Based on observations of cement, the greater structural formation for alkali-activated slag paste can be credited to the rapid polymerization and development of early reaction products. Furthermore, the addition of slag enhances the dissolution and condensation of blended mixes.

On the other hand, the dissolution of fly ash is mostly negligible as compared to that of slag within a specific time frame. Consequently, in blends of slag and fly ash, the slag portion contributes to structural development, and the spherical-shaped fly ash particles are effective for reducing interparticle friction and providing a lubrication effect. Moreover, the fly ash particles can provide additional nucleation sites for reaction products by a filler effect. Therefore, even low-reactive fly ash can enhance the reaction process of alkali-activated slag paste. It should also be noted that blended mixes present lower storage modulus values in a few cases, as fly ash enhances the workability, which delays the structural buildup of alkali-activated paste.

#### 3.4.2. Effect of Molarity

Figure 4 shows that alkali-activated mixes with higher molarity have higher initial storage modulus values. This is usually caused by the greater flocculation network due to the interparticle forces and to some extent the early-age reaction products. For later stages, the storage modulus of mixes with lower molarity levels register a greater increase. This increase can be due to the polycondensation phase of the reaction. It has been demonstrated that in blended mixes, fly ash particles act as nucleation sites, an effect which is enhanced as the molarity of the alkali-activating solution is increased.

#### 3.4.3. Effect of Temperature

It can be observed in Figure 4 that an increase in the temperature results in an increase in the storage modulus. Furthermore, an evident “spark point” with regard to the gain in the storage modulus can also be observed. The higher temperature results in the early onset of the spark point, leading to a rapid gain in the storage modulus. Previous work showed that heat curing is beneficial for strength development in alkali-activated mixes [47]. 

For alkali-activated fly ash paste, the change in the storage modulus at a temperature between 25 °C to 55 °C confirms the sluggish reactive rate of fly ash. At 55 °C, a significant improvement in the storage modulus is observed, which indicates the commencement of chemical reactions and the development of the initial reaction products. The increase in the temperature accelerates the dissolution of fly ash and the release of silicate and aluminate monomers. The precipitation of reaction products leads to the formation of sodium aluminate silicate hydrate (N-A-S-H) gel, which is evident given the rise in the storage modulus. For blended and slag mixes, a higher temperature shortens the time required for accelerating the gain in the storage modulus.

### 3.5. Loss Factor

The loss factor was evaluated to characterize the transition of the alkali activated paste to a solid state during the allotted measurement duration. The storage modulus (G’) is directly proportional to the energy stored and the loss modulus (G”) is directly proportional to energy the dissipated during the oscillation cycle. The loss factor is used to indicate viscoelastic behavior in an alkali activated paste [24]. A value of the loss factor equal to 1 indicates the “gel point”, at which point the storage modulus is equal to the loss modulus. If the loss factor exceeds 1, the paste is considered to be a viscoelastic liquid, whereas it is a viscoelastic solid at values of less than 1. This indicates that loss factors (<1) correspond to less viscoelastic solid behavior, which means that the material presents a dominant viscous deformation response.

**Figure 5:**
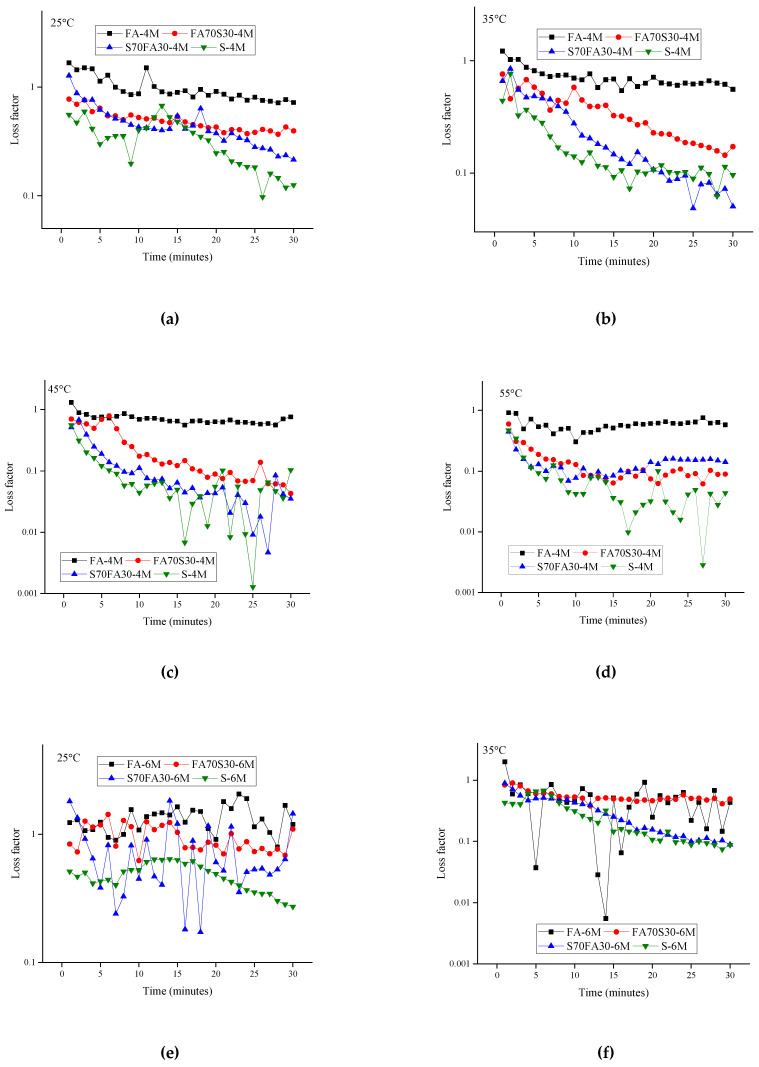

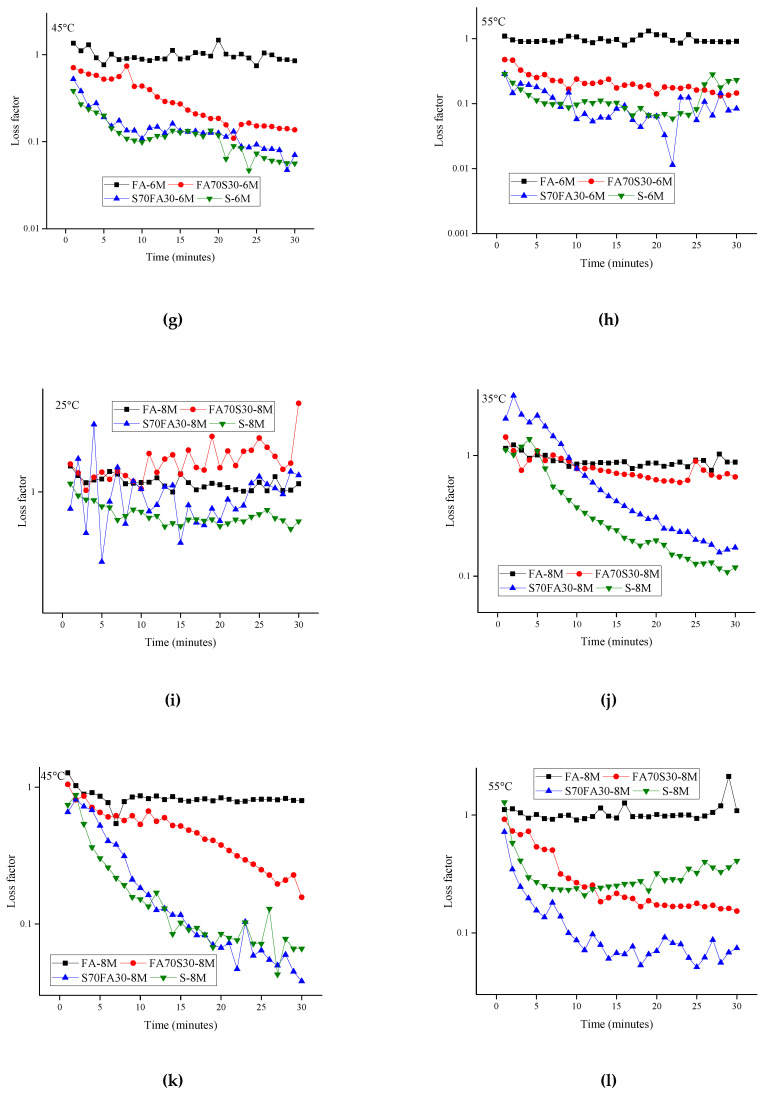

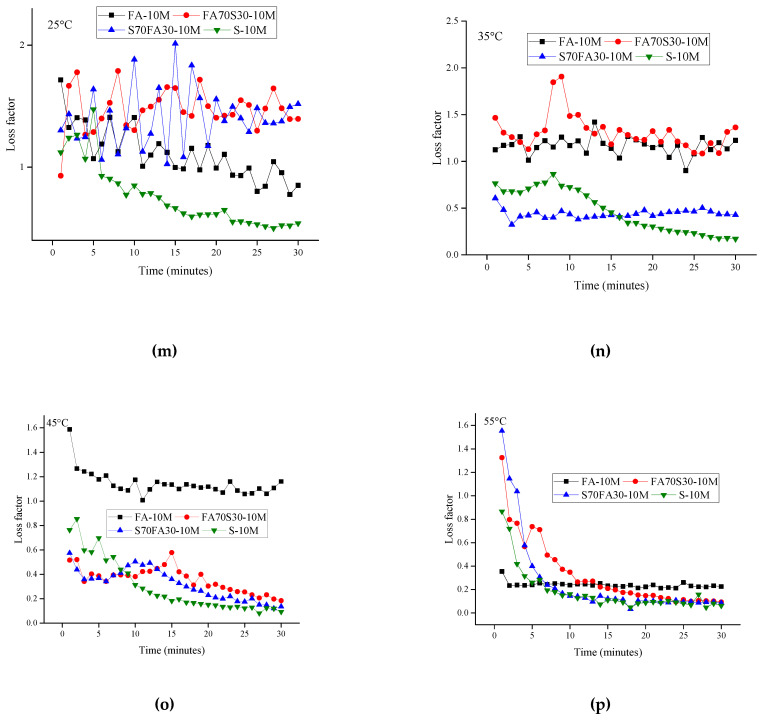
Evolution of loss factor with time for alkali-activated mixes: (**a**) 4 M at 25 °C; (**b**) 4 M at 35 °C; (**c**) 4 M at 45 °C; (**d**) 4 M at 55 °C; (**e**) 6 M at 25 °C; (**f**) 6 M at 35 °C; (**g**) 6 M at 45 °C; (**h**) 6 M at 55 °C; (**i**) 8 M at 25 °C; (**j**) 8 M at 35 °C; (**k**) 8 M at 45 °C; (**l**) 8 M at 55 °C; (**m**) 10 M at 25 °C (**n**) 10 M at 35 °C (**o**) 10 M at 45 °C; (**p**) 10 M at 55 °C.

Figure 5 presents the progress of the loss factor of the alkali-activated paste over time. The loss factor of alkali-activated mixes registers a decline over time. Gel formation over time is associated with a decline of the loss factor [48]. Furthermore, the small peaks and humps also represent gelation over a period of time [49]. Gelation refers to formation of polymerization products, such as C-A-S-H and N-A-S-H. The result indicates that the loss modulus energy is related to the relative motions of flocculated particles in the fly ash mixes due to the large particle size [22]. In addition, the alkali-activated slag and blended mixes achieve an elastic state in much less time as compared to the alkali-activated fly ash. The loss factor values of the slag mixes rapidly dropped without any peaks or humps, indicating less viscous deformation. For the alkali-activated mixes investigated at slightly higher temperatures (45–55 °C), the gradual decrease in loss factor represents a more rigid and elastic network for the alkali-activated matrix. Compared to the mixes at lower temperatures, the higher temperature reduces the lubricated particle contact, causing less energy loss. The mixes at higher temperatures showed a rapid decline in the loss factor in all cases up to a specific point of time, after which the values generally stabilized in a linear form, suggesting steady gel formation. The heat release of the alkali-activated mixes also has some effect on the loss factor of the alkali-activated mixes. Greater heat release for mixes with higher molarity levels leads to quicker gelation, as indicated by the reduced time required for the reduction in the loss factor.

## 4. Conclusions

The present study examines the influence of the precursor, the alkalinity of the alkali-activating solution and the temperature on the rheology and structural buildup of alkali-activated mixes. The following conclusions are drawn.

1. At 25 °C, the alkali-activated fly ash showed lower shear stress than the alkali-activated slag and blended mixes. The flocculation of slag particles and the increased interparticle forces resulted in shear stress of the slag and blended alkali-activated mixes. Moreover, the higher molarity of the mixes increases the repulsive forces between the precursor particles, leading to higher shear stress. The flow curve measurements showed that workability of alkali-activated materials was highly interdependent on multiple factors, such as precursor, molarity, etc.
2. As compared to alkali-activated fly ash, an increase in the viscosity of the alkali-activated slag was observed due to the higher dissolution of the precursor and the precipitation of the reaction products. Regarding the higher molarity of the alkali-activating solution, the effect of sodium silicate is diluted, which results in increased viscosity of the alkali-activated mixes. Furthermore, the viscosity measurements showed that particle size distribution also played an important role in higher viscosity values of alkali-activated slag.
3. The gain and degree of the storage modulus of the slag mixes were rapid when compared to those of the alkali-activated fly ash, which showed very poor structural buildup. The low dissolution of the fly ash was improved when the slag amount was increased in the mix. The storage modulus values provided critical insight in understanding how a precursor can affect the structural buildup of alkali-activated material.
4. The alkali-activated mixes with higher molarity of the alkali-activating solution registered a higher initial storage modulus due to a complex flocculated network. Higher molarity was instrumental in the rapid structural buildup due to the enhanced dissolution of the precursors and the precipitation of the reaction products. The measurement of storage modulus also showed that higher molarity resulted in higher nucleation sites.
5. An increase in the storage modulus was observed with a rise in the temperature. For the alkali-activated fly ash, a temperature above 35 °C enhanced the dissolution of fly ash particles and the precipitation of the reaction products. It can be stated that spark point of polymerization can be effectively suggested by using the isothermal rheology.
6. The loss factor was gradually reduced in the alkali-activated slag and blended mixes due to gelation and less viscous deformation. The alkali-activated fly ash showed small peaks and humps in the loss factor, which indicated a higher loss modulus over the storage modulus. The higher molarity and temperature of the alkali-activated mix showed a rapid reduction in the loss factor values due to the greater amounts of reaction products, which indicated a viscoelastic solid phase. The loss factor measurement was quite instrumental in understanding the steady gel formation of alkali-activated materials.

## Figures and Tables

**Table 1:** Chemical composition of fly ash and slag used in this study.

Oxide Composition	Fly Ash (wt.%)	Slag (wt.%)
SiO_2_	58.88	28.47
CaO	6.32	39.71
Al_2_O_3_	24.50	17.53
Fe_2_O_3_	6.88	0.91
SO_3_	0.74	1.54
MgO	1.64	11.12
Na_2_O	0.50	0.14

**Table 2:** Yield stress and plastic viscosity of alkali-activated mixes.

Mix	Yield Stress (Pa)	Plastic Viscosity (Pa.s)
FA-10M	16.05	6.15
70FA30S-10M	18.23	7.43
70S30FA-10M	25.87	11.76
S-10M *	-	-
FA-8M	4.50	1.5
70FA30S-8M	6.54	2.83
70S30FA-8M	10.50	9.53
S-8M *	-	-
FA-6M	4.37	1.29
70FA30S-6M	6.19	1.98
70S30FA-6M	16.05	6.15
S-6M	20.82	9.35
FA-4M	4.35	2.06
70FA30S-4M	5.43	2.08
70S30FA-4M	6.13	5.5
S-4M	16.55	8.70

* The minimum values required for the calculation was not achieved due to rapid solidification.

**Table 3:** LVER limit, LVER proposal and storage modulus of alkali-activated mixes.

Mix	Shear Strain (%)	Shear Stress (Pa)	Storage Modulus (Pa)
FA-10M	0.01	0.015	74.50
70FA30S-10M	0.01	0.007	33.33
70S30FA-10M	0.01	0.007	29.90
S-10M	0.01	0.007	32.46
FA-8M	0.01	0.007	37.49
70FA30S-8M	0.01	0.006	42.87
70S30FA-8M	0.01	0.008	48.11
S-8M	0.01	0.003	15.59
FA-6M	0.01	0.003	17.38
70FA30S-6M	0.01	0.004	22.87
70S30FA-6M	0.01	0.006	34.68
S-6M	0.01	0.003	26.72
FA-4M	0.01	0.015	143.30
70FA30S-4M	0.01	0.014	128.30
70S30FA-4M	0.01	0.004	35.34
S-4M	0.01	0.006	46.31

## Data Availability

The data presented in this study are available on request from the corresponding author.
